# Age polyethism can emerge from social learning: A game-theoretic investigation

**DOI:** 10.1371/journal.pcbi.1013415

**Published:** 2025-08-25

**Authors:** Moein Khajehnejad, Julian García, Bernd Meyer

**Affiliations:** 1 Department of Data Science and Artificial Intelligence, Faculty of Information Technology, Monash University, Clayton, Victoria, Australia; 2 Turner Institute for Brain and Mental Health, Monash University, Clayton, Victoria, Australia; Interdisciplinary Transformation University IT:U, AUSTRIA

## Abstract

Age-polyethism—the age-based allocation of tasks in social insect colonies—is a key feature of division of labour. While its hormonal underpinnings have been studied extensively, the behavioural and environmental mechanisms driving age-polyethism remain poorly understood, especially under ecological stress. We present a novel modelling framework that integrates social learning with task-related environmental feedback to explain the emergence and breakdown of age-polyethism. We develop two models: a Social Learning (SL) model, in which individuals adapt task preferences by copying similar peers, and a Stimulus-Response Threshold Social Learning (SRT-SL) model, which extends this framework by incorporating task-related dynamic stimuli and response thresholds that regulate collective task demand. Our models demonstrate that age-polyethism can emerge from simple social imitation processes, without the need for fixed hormonal schedules. We show that under increasing environmental pressure (e.g., resource scarcity), age-polyethism collapses as younger individuals are forced into tasks typically handled by older workers. Importantly, we find that age-polyethism does not necessarily optimize immediate colony efficiency; instead, it appears to reflect a trade-off between environmental constraints and behavioural coordination. These findings provide a mechanistic and ecologically grounded explanation for empirical observations linking environmental stress to dysfunctional division of labour and colony collapse.

## Author summary

In many social insect colonies, like those of bees, workers perform different task sets at different ages (age polyethism). Commonly, younger individuals perform safe tasks inside the nest while older ones take on risky jobs like foraging. This age-based division of labour is often attributed to internal hormonal schedules, but how social behaviour and environmental stress affect this system is poorly understood. We developed a computer model to investigate these factors. In our model, individuals learn which tasks to perform by imitating other workers, paying more attention to their age peers. We then introduced environmental pressures to see how the self-organised division of labour would adjust. Our model shows that this simple form of social learning is enough for an age-based workforce structure to emerge that still maintains flexibility, without the requirement for a rigid internal clock. Crucially, when we applied strong environmental stress, this system collapsed and younger workers were forced into dangerous foraging tasks prematurely, which is consistent with empirical results. We also found that this division of labour may not be about maximizing day-to-day efficiency, but rather about ensuring long-term colony stability and resilience. These findings provide a new, mechanistic explanation for age polyethism and how environmental pressures can disrupt the social organization of a colony, offering key insights into the real-world problem of colony collapse.

## 1. Introduction

Social insect colonies are among the most successful collectively living organisms. Their efficient division of labour (DOL) is a key factor for this success [1]. Rather than the division between reproductives and non-reproductives, we are interested in how different workers specialize within a colony to perform specific tasks, such as foraging, nest constriction, brood care, defense, etc. An important aspect of DOL is that task allocation can often be flexibly adjusted [2,3]. In social insects, DOL often occurs in the form of *age-polyethism*, meaning that the age of individuals determines the task sets they can perform [4].

As an example, honeybees devote their youngest workers to cleaning cells. From 2 to 11 days of age, they advance to duties related to brood care and nest maintenance. From days 11 to 20, they receive and store food collected by foragers, and around day 20, they begin foraging [5]. There are similar patterns of temporal polyethism in primitive species of wasps, such as *Ropalidia marginata* as well as the eusocial wasp *Vespula germanica* [6]. Young workers start out feeding larvae, before transitioning to nest-building duties and then foraging. These patterns are also exhibited by a number of species in ants [7]. Generally, a division where younger workers perform predominantly in-nest tasks, whereas older workers graduate to out-of-nest tasks is common. There is no rigidity to this pattern, however. There is a strong tendency for workers of a certain age to perform specific tasks, but they may choose to perform other duties if enough need arises. For example, foragers, especially younger foragers, will revert to nest-care tasks when young in-nest workers are removed from the nest [8]. It has been empirically confirmed that age-polyethism does not guarantee efficiency maximization; in some species of ants, older workers are found to be more effective at caring for brood than younger ones [8]. Instead, the most hazardous tasks are entrusted to workers with the lowest remaining life expectancy. The division of labour in honeybees described above is an instance of this phenomenon. Similarly, injured workers who have a shorter life expectancy will begin foraging earlier than healthy workers [9].

Colonies can suffer serious viability problems due to age-polyethism dysfunction and environmental stress factors are empirically related to such failures [10–12]. In response to environmental stress, the task-age structure may change, causing younger workers to become precocious foragers with higher mortality rates and poor performance. These dynamics can lead to rapid colony decline and collapse [10]. This study offers a strong argument that a detailed understanding of how task allocation dynamics operate can significantly contribute to our ability to diagnose the circumstances under which colonies may be at risk. While it is empirically well-established that environmental stress can reshape age-polyethism and lead to colony instability [10,30], the underlying behavioural mechanisms responsible for this breakdown remain poorly understood. Previous models have captured these effects at a macro or demographic level [10], but they have not addressed how individual-level learning and interactions give rise to these emergent colony dynamics. Our model is constructed to fill this gap by explicitly linking environmental stress, individual social learning, and the resulting collective patterns of task allocation.

Age-polyethism has so-far primarily been investigated empirically and there is only a limited amount of theoretical modelling work [10,13]. Hormonal changes throughout life have empirically been identified as one of the important drivers [14,15]. However, there is also evidence that the age at which individuals begin to switch tasks remains constant regardless of hormonal changes [16] and other results likewise hint that hormone-related explanations alone may not capture the full picture [17].

Generally, it is well established that task allocation is frequently influenced by environmental factors such as food availability, predation threats, and climate conditions [18,19]. Moreover, the social context and the interactions of individuals are relevant to the task selection process according to several empirical [20–22] and theoretical studies [23]. It is thus worthwhile asking whether other drivers, in particular environmental and behavioural ones, may exist and, specifically, whether these may explain the collapse of age-polyethism in the presence of environmental stress.

We advance an alternative possible explanation for age-polyethism—that age-related specialisation may at least partly arise from social learning because individuals are more likely to imitate others who are alike in attributes such as morphological factors. We construct a mathematical model of learning in a colony that integrates social interactions and environmental factors and provides a detailed view of how age polyethism can emerge from social learning.

Our model is motivated by the empirical knowledge that insects learn not only through their own experience but also from others’ behaviours [24], and specifically by recent experimental breakthroughs that demonstrate instances of social learning mechanisms in social insects that are far more intricate than previously thought [25,26]. Given the fact that physiological explanations do not fully explain age-polyethism, we investigate whether social learning of this type can be a proximate mechanism for age-polyethism. While one of the best-established models for learning in social insect colonies are response threshold models [27,28], these models do not account for social learning because they model behaviour as a reaction to environmental stimuli only, not as an outcome of interactions among individuals. They lack a mechanism for individuals to adapt their task preferences based on direct interaction with others and can only capture indirect interactions via stigmergy. Our modelling framework is thus based on the Evolutionary Game Theory [29] that explores how individuals adapt to their interactions with others as well as their surroundings.

It is important to note that, despite the use of the established terminology “evolutionary” game theory (EGT), this paper is concerned with behavioural dynamics on colony lifetime scales rather than evolutionary aspects. Here, we use the EGT framework to investigate the dynamics of a learning process in colony lifetime. We could thus refer to the framework as “learning game theory”. However, we prefer to adhere to the standard terminology to avoid confusion.

To examine how social learning can contribute to age-based division of labour, our model considers a simplified setup with two age groups and two task types—an out-of-nest maximising task and an in-nest homeostatic task. This minimal structure is sufficient to capture key dynamics of age-polyethism under varying environmental conditions; Further elaboration is provided in the Discussion.

Based on our model we show why and how environmental factors can shape the dynamics of age groups via the experience of task execution, for example, task difficulty.

The main contributions of the present paper can thus be summarised as follows: (1) We provide a new, alternative explanation for the proximate mechanisms of age-polyethism, namely that it can arise from social learning and present a detailed mathematical model for this. (2) We show that age polyethism can be reshaped by environmental pressures and provides a plausible explanation for colony breakdown under environmental pressure.

Furthermore, we confirm that age-polyethism does not always lead to the highest colony efficiency.

## 2. Materials and methods

Behavioural response threshold models are well-established and commonly employed to investigate DOL [27,28]. Repsonse threshold models have a simple and elegant structure. They assume that individual workers have an internal threshold for task-related stimuli and that they will react to a task demand based on the relationship between this threshold and the currently perceived stimulus level. This general idea has been used in many different forms. Arguably, the most widely used model is the so-called reinforced response threshold model, in which the dependency of the response probability on the stimulus level is modeled as a sigmoid function, for example:

$$p_{\theta }(s)=\frac{s^{2}}{s^{2}+\theta^{2}},$$ (1)

where *s* is the stimulus intensity and *θ* is the internal response threshold [31]. *θ* is assumed to either be fixed [31] or vary over time [27] in response to task execution. For the latter case, *θ* can, for example, be given as:

$$\theta (t+1)=\left\{ \begin{array}{cc} \theta (t)-\xi & \text{if the task is performed at time t};\\ \theta (t)+\varphi & \text{otherwise}, \end{array}\right.$$ (2)

where ξ and φ give the speeds of learning and forgetting respectively [27,32].

While response threshold models have explained a number of fundamental aspects of DOL, there is now growing evidence that response thresholds alone cannot explain all empirical patterns of DOL in social insects [33,34], specifically because they do not directly account for individual interactions. It is, however, well known that social context and direct interactions play a significant role in task allocation [19,35,36]. An alternative approach is to employ Evolutionary Game Theory (EGT) to capture individual interactions and model how individuals in a population learn to act as a group by continuously adjusting their bevaviours [29,37,38].

Game theory offers a mathematical framework that directly accounts for the cost of producing a common good. Environmental factors, such as the difficulty of foraging for individuals belonging to different age groups, can thus be immediately included in a behavioural model. We reiterate that while the application of game-theoretic models to animal groups has originally focused on the evolutionary origin of behaviours [39], our work is concerned with the learning of behaviours: rather than investigating how (genetic) traits propagate in reproduction, we ask how behaviours propagate by social learning.

Here, we develop a *Stimulus-Response Threshold Social Learning (SRT-SL)* model integrating a response threshold approach with social learning based on an EGT framework. We start by formalising a purely social learning model based on EGT (termed the “SL model”, cf. Sect 2.1) that captures how agents learn from each other in direct interactions but that does not yet account for stigmergy via the task-related stimulus. We then extend this model to also capture the stygmeric interaction via the task stimulus in much the same way as response threshold models do (termed the “SRT-SL” model, cf. Sect 2.2).

Despite the fact that this starts from a perspective of social learning, this framework is also compatible with and can in the limit be seen as an extension of the well-established response threshold model [27].

Prior to describing our models in detail, we briefly recapitulate the evolutionary game theory framework. Consider a finite population *P* of agents, each fully characterized by a set of trait values. Here, the term *trait* does not refer to a genetic trait but to an abstract property that captures relevant aspects of its behaviour. The model evolves in discrete time steps. At each step, agents interact in fixed-size groups of *n* (“*n*-player games” in EGT), and each agent *i* receives a payoff $\Pi_{i}$ from the interaction. Π generally depends on agent *i*’s own trait values as well as those of the other agents in the game. Agents learn from each other by adopting the trait values (i.e. behavioural characteristics) of another agent. How strongly a trait value propagates (i.e. the probability of it being copied) is determined by the success of the individuals that carry this trait value: successful individuals are more likely to be copied. Note that this can be interpreted as recruitment as well as other forms of entrainment, including imitation. If the recruitment effort is modulated by task performance experience, successful agents will be more readily imitated.

Our model describes the dynamics of task engagement for a given age structure of the population. In other words, it describes the fast dynamics of task engagement that a given age structure induces rather than the slow change due to the aging process of the colony. We focus on a modified version of recruitment that captures our core-assumption of age-dependent recruitment. We assume that the recruitment signal is modulated by (1) task performance experience; meaning that successful agents perceiving higher payoffs will recruit more strongly; and (2) similarity between individuals, meaning that individuals let themselves be recruited more readily by others they are similar to. The latter could simply arise from the fact that similar individuals interact more frequently, for example, due to the spatial separation of in-nest and out-of-nest contexts. It also accounts for the possibility that individuals are more likely to imitate other individuals that are morphological more similar to themselves.

We start with a task allocation problem that only involves the choice between two prototypical tasks. The general characteristics of these two tasks and the environment are captured in the shape of the cost and benefit functions that together determine the payoff Π. We stress that costs and benefits do not directly reflect a fitness benefit (as this would be on the colony level) but instead a direct task performance experience that may cause individuals to engage more or less with the task from which the experience arises [34].

For the purpose of our discussion, we pick two task types with contrasting characteristics: *(i)* a task with a linear benefit shape and *(ii)* a task with a concave benefit shape. Foraging is a good example for the first type (Task 0): benefits increase as engagement levels increase. Regulatory homeostatic tasks, such as thermoregulation, are good examples of the second type (Task 1): a certain amount of effort is required to keep the colony at the optimal temperature and the optimum benefit is obtained when this effort is invested. Further effort does not improve the benefit and may, in fact, even decrease it; for example due to overcooling by fanning or overheating by incubation [40].

We assume a marginally decreasing cost function for the first task and a linear cost function for the second. This reflects the fact that the first task commonly becomes easier with practice, i.e. when more effort is invested. In addition, we assume that generally, the first task is easier (less costly) for *old* individuals compared to *young* ones. Note, however, that this is not universally true for all tasks in all species [41]. There are many other function shapes relevant to other task types which can be examined similarly. For the purpose of establishing the general framework, we shall focus on these two types.

Additionally, as we are interested in how environmental influences act on task allocation in different age groups, we introduce three further parameters: *b* and *r* that link cost and benefit to the characteristics of the environment, and *β* which determines the cost difference of Task 0 between *young* and *old* individuals. Parameter *b* captures the ratio between the benefit and cost of Task 0. Larger values of *b* represent that foraging is cheap (abundant environment), whereas small values of *b* indicate that foraging is more costly (food scarcity). Similarly, *r* is the cost ratio of Tasks 1 and 0.

The parameter *β* is specifically applied within the cost function for Task 0, $C_{0}(x_{j},a_{j})$, as shown in Eq 3:

$$C_{0}(x_{j},a_{j})=(-(1-x_{j}{)}^{2}+2\;(1-x_{j}))\cdot \exp(\beta \;(1-a_{j})).$$ (3)

Here, *x*_*j*_ represents individual *j*’s engagement with Task 1, $1\;-\;x_{j}$ their engagement with Task 0, and *a*_*j*_ their age. The term $\exp(\beta (1\;-\;a_{j}))$ directly scales the cost of Task 0 based on age. As *a*_*j*_ (age) increases, $\left(1\;-\;a_{j}\right)$ decreases, leading to a smaller exponent and thus a lower cost for older individuals. Conversely, for younger individuals (smaller *a*_*j*_), $\left(1\;-\;a_{j}\right)$ is larger, resulting in a higher exponent and a greater cost. Therefore, *β* controls the magnitude of this age-dependent cost difference; a higher *β* value implies a steeper increase in foraging cost for younger individuals compared to older ones.

**Fig 1:**
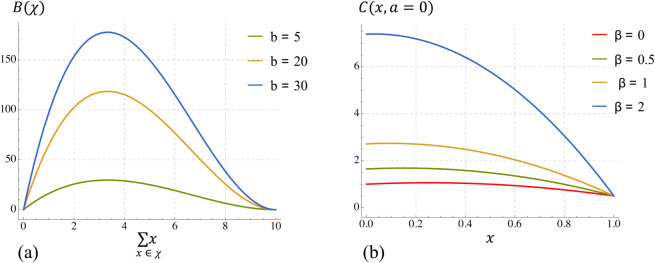
Exploring the shapes of a) Total Benefit and b) Total Cost functions from Eq 12 in Appendix 1 with respect to various b and *β* values. Note that in *b*), the total cost tolerated by a given *young* individual is illustrated for different *β* parameters. Hence, the curve corresponding to β=0 is equivalent to the cost borne by any *old* individual.

The shapes of the benefit and cost functions are shown in Fig 1 and full details are given in Appendix 1.

We start from a simple core model of social learning (SL model) that does not yet take into account that response probabilities can depend on the strength of a task-related stimulus. This is added to the model in a subsequent step to build the complete stimulus-response threshold social learning (SRT-SL) model. In this extended model, individuals’ decisions to perform tasks are influenced by both learned social information and their individual responses to task-specific environmental cues (stimuli), where a task is only initiated if the stimulus exceeds an individual-specific threshold.

### 2.1. *Social Learning (SL)* model

We divide the population into two separate age groups, namely *young* and *old*. Each worker *i* is completely characterised by a tuple, $\left(x_{i},a_{i}\right)$, where formally, $x_{i}\in R$ is the probability to execute the first task, $1\;-\;x_{i}$ the probability to execute the second task, and $a_{i}\in \{0,1\}$ is an indicator of the age group. Note that the execution probability of tasks will be made dependent on task-related stimuli in the following section. A population of *N* workers is thus given by a two-dimensional vector of trait values $(x_{j},a_{j}{)}_{j=1,\cdots ,N}$. We assume that task choices occur on a significantly faster time scale than trait changes, so that the trait value *x* can also be interpreted as the average fraction of effort invested into the first task (1–*x* is for the second task). As standard in EGT, the population is divided into *K* groups of interacting players (“*n*-player games”) $G_{1},G_{2},\ldots ,G_{K}$, where $K=\frac{N}{n}$.

Next, we specify the payoffs. Benefits in social insect colonies are shared and depend on the collective invested effort. Another key feature is that both tasks need to be performed by an appropriate number of workers for the colony to function well; benefits thus depend on both tasks simultaneously. We model this with a multiplicative coupling of benefit *B*_0_ of Task 0 and *B*_1_ of Task 1.

$$B({𝒫}_{k})=\frac{1}{n}\;B_{0}(\sum_{x\in {𝒳}_{k}}x)\cdot B_{1}(\sum_{x\in {𝒳}_{k}}x).$$ (4)

Here $\sum_{x\in {𝒳}_{k}}x$ is the collective effort invested by all individuals in *G*_*k*_ into the first task. It is important to stress that in generating the shared benefit, it is the collective effort, not the individual’s age, that plays a role.

Engaging in a task incurs costs that are borne individually. These can be thought of as energy expenditure. These costs depend on both the effort invested as well as on the individual’s age (reflecting more efficient task execution with experience and, potentially, morphological suitability). Costs for multiple tasks are naturally additive.

$$C(x_{j},a_{j})=C_{0}(x_{j},a_{j})+C_{1}(x_{j}).$$ (5)

Here, the cost of the foraging task $C_{0}(x_{j},a_{j})$ is age-dependent while the cost of the thermoregulation task $C_{1}(x_{j})$ only depends on the invested effort (see Appendix 1). The total payoff for each individual is the difference between its share in the collective benefit and its individual task performance cost. In game *G*_*k*_ individual *j* thus receives a payoff $\Pi_{j,G_{k}}$ as:

$$\Pi_{j,G_{k}}=\;B({𝒳}_{k})-C(x_{j},a_{j}).$$ (6)

In our framework, costs and benefits are defined at the level of the individual, as they represent task performance feedback that directly shapes behavioural adaptation. Since individuals are the active agents of change, it is their perceived experience, rather than colony-level consequences, that drives the learning dynamics.

**Fig 2:**
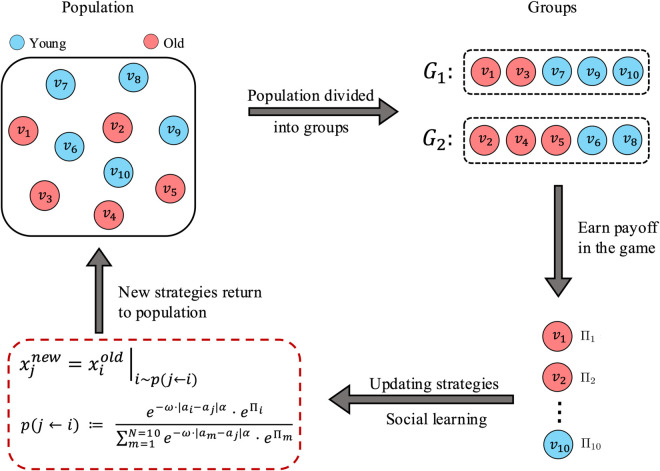
Schematic diagram of different steps in the social learning paradigm of the SL model. The social learning paradigm is followed similarly to Fig 4. After payoff calculations in each game (*G*_1_, *G*_2_), individual strategies (i.e. trait value or *x*) are updated. The updated population with the new trait values enters the next iteration in a loop until the steady state is reached.

Fig 2 depicts the overall schematic of the dynamic process of the system. We follow the standard approach to EGT simulation in which individual games are sampled from a larger population [43].

At each time step, the population is randomly divided into *K n*-player games and all individuals receive payoffs according to their trait values and the composition of the interaction groups they are participating in. When an individual *j* is recruited by another individual *i*, it changes its strategy to *i*’s strategy. This happens with a probability that depends on the success (payoff) of *j* relative to the population average.

$$p(j\leftarrow i)=\frac{e^{-\omega \cdot |a_{i}-a_{j}|\alpha }\cdot e^{\Pi_{i}}}{\sum_{m=1}^{N}e^{-\omega \cdot |a_{m}-a_{j}|\alpha }\cdot e^{\Pi_{m}}},$$ (7)

where *ω* is a constant and α∈[0,1] controls the strength of recruitment from the other age group. α=0 means that individuals disregard similarity when imitating and the probability of imitating individuals from the opposite group decreases with increasing *α*. This is equivalent to the recruitment strength in [37] with an added term to modulate the strength by age similarity.

It is also possible for trait values to undergo autonomous minor variation; that is individuals can explore the behaviour space by making small, random adjustments to their strategies (a form of slight behavioural plasticity), independently of social learning: $x_{j}^{t}\leftarrow 𝒩(x_{j}^{t},\sigma )$.

### 2.2. *Stimulus-Response Threshold Social Learning (SRT-SL)* model

The above pure social learning model captures the interactions between individuals, but it neglects the stigmergic effects that are the main component mediating the collective regulation in response-threshold models. That is to say, in response threshold models, the actions of each individual change the demand for its adopted task and thus the corresponding task-related stimulus to which other individuals react in turn.

We thus extend the model by adding a task-related stimulus *S*_*i*_ for each task *i* and reinterpreting the first component *x*_*i*_ of the trait tuple as a stimulus threshold to execute the first task rather than as a probability. As the threshold for a task increases, the propensity to engage in it diminishes (given a fixed stimulus level).

**Fig 3:**
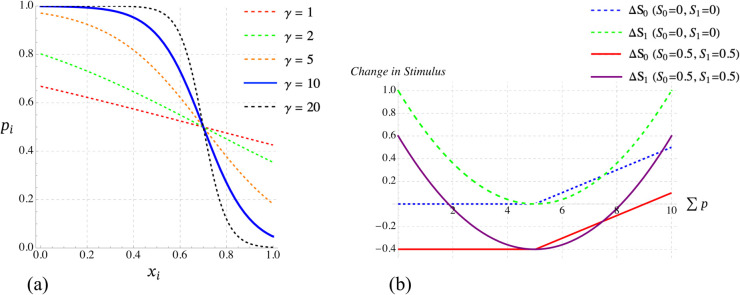
a) The shapes of the probability function *p*_*i*_ of individual i adopting Task 1 with respect to *x*_*i*_ for a set of different *γ* values and a fixed $S_{1}-S_{0}=0.2$ (see Eq 8). The selected *γ* value to get the results of this manuscript is shown in solid blue line. *b*) The shapes of changes in Task 0 and Task 1 stimulus values with respect to Σp for fixed values of group size, $B_{0_{\text{min}}}$ given *b* = 20, and $B_{1_{\text{max}}}$ (see Eqs 9, 10, and S1 Table in Appendix 1 for the choice of parameters) and two different initial values for *S*_0_ and *S*_1_. The selected parameter values used in our experiments below are shown in solid lines.

Concretely, the probability *p*_*i*_ for individual *i* to adopt Task 1 is a function of its threshold and the task-related stimulus levels.

$$p_{i}=1-\frac{1}{1+e^{-\gamma [(x_{i}-0.5)+(S_{0}-S_{1})]}},$$ (8)

where *γ* controls the slope of the function (Fig 3a).

For the maximising Task 0, the stimulus should stay constant if more than a certain amount of work is done and grow if less (not enough) work gets done. For the homeostatic Task 1, the dynamics of the stimulus should be such that it stays constant if a particular amount of work is done and grows if less work is done. The following stimulus dynamics captures this (see also Fig 3b):

$$\Delta S_{0}=\lambda_{1}\cdot max(0,B_{0_{min}}-\sum_{i=1}^{K}[B_{0}(\sum_{p\in {𝒫}_{i}}p)])-\lambda_{2}S_{0},$$ (9)

$$\Delta S_{1}=\phi_{1}\cdot (B_{1_{max}}-\sum_{i=1}^{K}[B_{1}(\sum_{p\in {𝒫}_{i}}p)])-\phi_{2}S_{1},$$ (10)

where $B_{0_{min}}$ is the overall minimum benefit acquired in the foraging task from all the groups which is necessary to avoid regulation failure. $B_{1_{max}}$ is the maximum possible shared benefit that the total sum of games can produce from thermoregulation.

For simplicity, our model assumes that at least half of the workforce must be engaged in foraging to resolve the associated demands. $\lambda_{1}$ and $\phi_{1}$ are normalizing parameters to keep the stimuli values in the unit interval.

The shapes of the probability function *p*_*i*_ of individual *i* adopting Task 1 with respect to *x*_*i*_ for a set of different *γ* values (Eq 8), as well as the shapes of changes in Task 0 and Task 1 stimulus values with respect to Σp for fixed values group size, $B_{0_{\text{min}}}$, and $B_{1_{\text{max}}}$ (Eqs 9, 10, and S1 Table in Appendix 1), and two different initial values of *S*_0_ and *S*_1_ are shown in Fig 3.

**Fig 4:**
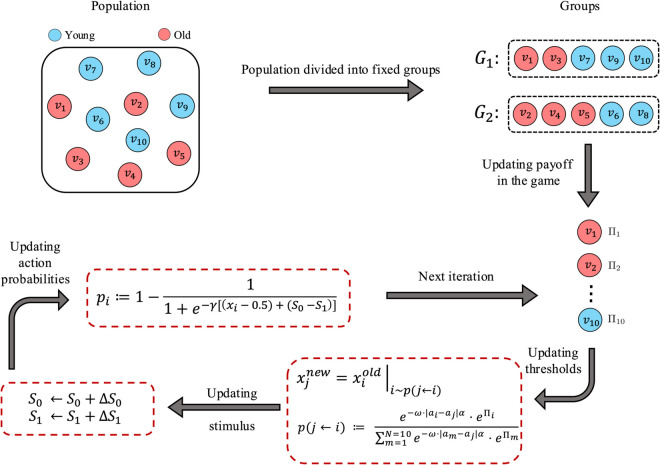
Schematic diagram of different steps in the modified *SRT-SL* framework. Each coloured circle represents a *young* or *old* individual in the colony. In the first step of the evolutionary process, the population is divided into different games (*G*_1_ and *G*_2_). The payoffs of each individual are then obtained in the corresponding games and the calculated payoffs form the basis for choosing successful individuals to copy. This step is then followed by an update in individual thresholds (i.e. trait value or *x*) and task stimuli (i.e. task demands or *S*). This step is then followed by the update of action probabilities, *p*_*i*_, based on the new stimulus functions in the colony. Stimulus functions for both tasks (*S*_0_ and *S*_1_ change by an amount equal to the sum of stimuli variations in all the games. The updated population with the new trait values enters the next iteration in a loop until the steady state is reached.

Fig 4 and Algorithm 1 represent the details of this modified version of the model or *SRT-SL*.

**Algorithm 1 Stimulus-Response Threshold Social Learning (SRT-SL): At each generation *t*, each individual *j* updates its threshold $x_{j}^{t}$ following an imitation phase and a mutation with probability *μ* as well as its action probability $p_{j}^{t}$.**

**Require:** A population of size *N* with a strategy profile $Q^{0}=\{(x_{1}^{0},a_{1}),(x_{2}^{0},a_{2}),\cdots ,(x_{N}^{0},a_{N})\}$ at generation *t* = 0; $\left(x_{j}^{0},a_{j})=(x_{0},a_{0})\in ([0,1],\{0,1\}\right)$; initial stimulus levels, $\left\{S_{0}^{0},S_{1}^{0}\right\}$; age group selection intensity, *α*; age similarity index tuning parameter, *ω*; mutation rate, *μ*; standard deviation for Gaussian mutations, *σ*.

1: generate K=Nn random fixed games and partition individuals into the games of size n

2: Q←[ ]

3: P←[ ] 4: **for** j=1:N **do**

5:   $p_{j}^{0}\leftarrow 1-\frac{1}{1+e^{-\gamma [(x_{j}^{0}-0.5)+(S_{0}^{0}-S_{1}^{0})]}}$

6: **end for**

7: $P^{0}\leftarrow \{p_{1}^{0},p_{2}^{0},\cdots ,p_{N}^{0}\}$

8: **for** t=1:T **do**

9:   *Q*.*append*(*Q*^*t*−1^) , *P*.*append*(*P*^*t*−1^)

10:   **for** j=1:N **do**

11:    $\Pi_{j,G_{k}}\leftarrow B({𝒫}_{k}^{t-1})-C(p_{j}^{t-1},a_{j})\text{ where }j\in G_{k};\;k\in \{1,\cdots ,K\}$

12:   **end for**

13:   **for** j=1:N **do**

14:    $\text{randomly chooses a parent }i\text{ to imitate with probability }\frac{e^{-\omega \cdot |a_{i}-a_{j}|\alpha }\cdot e^{\Pi_{i}}}{\sum_{m=1}^{N}e^{-\omega \cdot |a_{m}-a_{j}|\alpha }\cdot e^{\Pi_{m}}}$

15:    $x_{j}^{t}\leftarrow x_{i}^{t-1}$

16:   **end for**

17:   **for** j=1:N **do**

18:    **if** random()<μ **then**

19:     $x_{j}^{t}\leftarrow max(0,min(𝒩(x_{j}^{t},\sigma ),1))$

20:    **end if**

21:   **end for**

22:   $S_{0}^{t}\leftarrow S_{0}^{t-1}+(\Delta S_{0}{)}^{t-1}$ , $S_{1}^{t}\leftarrow S_{1}^{t-1}+(\Delta S_{1}{)}^{t-1}$

23:   **for** j=1:N **do**

24:    $p_{j}^{t}\leftarrow 1-\frac{1}{1+e^{-\gamma [(x_{j}^{t}-0.5)+(S_{0}^{t}-S_{1}^{t})]}}$

25:   **end for**

26:   $Q^{t}\leftarrow \{(x_{1}^{t},a_{1}),(x_{2}^{t},a_{2}),\cdots ,(x_{N}^{t},a_{N})\}$

27:   $P^{t}\leftarrow \{p_{1}^{t},p_{2}^{t},\cdots ,p_{N}^{t}\}$

28: **end for**

29: **Return** $Q=\{Q^{0},Q^{1},\cdots ,Q^{T-1}\}$ , $P=\{P^{0},P^{1},\cdots ,P^{T-1}\}$

## 3. Results

We implemented both models as individual-based, discrete-time simulations. All simulations were implemented twice independently for verification purposes, once in Python 3.7.3 and once in Wolfram Mathematica 12.3.

We find that age-polyethism emerges in the system under certain conditions and that the crucial determinants for this to happen are the difference in task difficulty for different age groups as well as the extent of imitation capability between different age groups.

Appendix 2 shows that our simulation results agree with a theoretical analysis based on *adaptive dynamics* [42,43], an approach that is widely used to analyse the dynamics of evolutionary game theory models under the assumptions of infinite populations and small mutations.

### 3.1. Behavioural regions in the environment and emergence of age-polyethism

We analyse how the simulation results depend on parameters that describe environmental characteristics (see S1 Table in Appendix 1): *b* scales the relative benefit obtained for one unit of work from foraging, i.e. it relates to the abundance of the environment. *β* captures the cost difference of foraging between young and old workers, with higher values indicating greater costs for young agents. *α* controls the strength of recruitment between different age groups: α=0 means that there are no restrictions and the probability to imitate individuals from the other age group decreases with increasing *α*. Finally, *r* measures the relative cost between the two tasks. The population is evenly split into young and old individuals.

**Fig 5:**
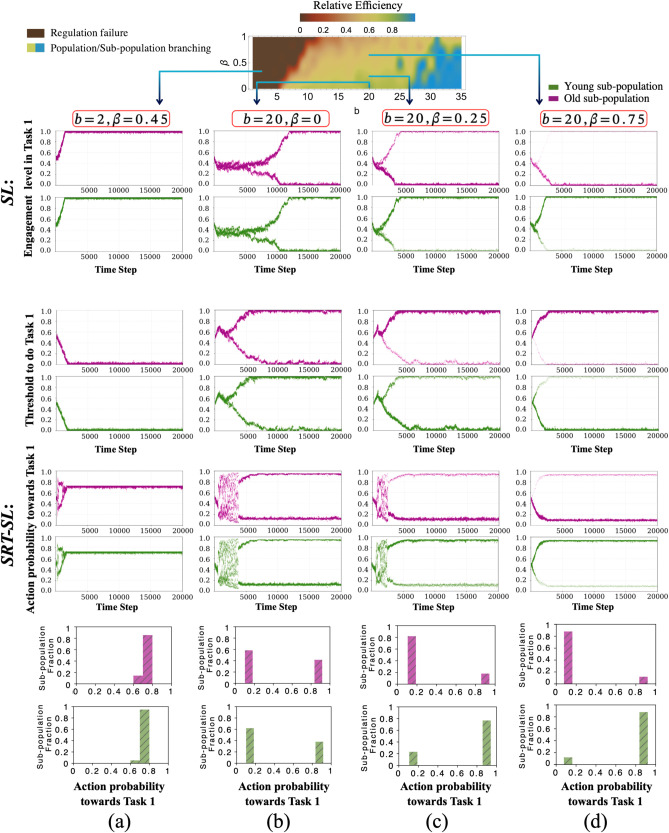
Relative colony efficiency and simulation results for α = 0.5 in different behavioural regions when varying β and b for the SRT-SL and SL model. The simulation results of the ***SL*** model represent the evolution of engagement levels in Task 1 among the *young* (green) and *old* (purple) sub-populations. Engagement levels are defined as the fraction of the subpopulation engaging in Task 1. The simulation results of the ***SRT-SL*** model show the evolution of worker thresholds for Task 1 for the *young* (green) and *old* (purple) sub-populations. The simulation results of the ***SRT-SL*** model are also represented to show the evolution of action probabilities towards Task 1 among the *young* (green) and *old* (purple) sub-populations. The histograms illustrate the fraction of individuals at different levels of action probability towards Task 1 for *young* and *old* sub-populations in green and purple, respectively. Bar widths reflect chosen bin sizes for visualization. The simulations correspond to different environmental settings: **a)** *b* = 2, β=0.45; **b)** *b* = 20, β=0; **c)** *b* = 20, β=0.25; **d)** *b* = 20, β=0.75.

We start by investigating a system with α=0.5. Fig 5 shows the results for both the *SRT-SL* model and the *SL* model. Fig 5 also illustrates the *relative population efficiency* measured as the ratio between the average payoff achieved and the maximal payoff achievable with optimal workforce allocation (closely related to the *price of anarchy* [44]). Here, we determine the optimal workforce allocation using Simulated Annealing, a stochastic global-optimisation metaheuristic [45].

The most interesting insight overall is that in both models, age-polyethism can emerge under specific conditions (Figs 5c-d). However, this division of labour collapses under increasing environmental pressure (Fig 5a, which shows decreased abundance *b* = 2) or when the age-dependent cost differential for foraging is lacking (Fig 5b). This is clearly visible in the subplots showing engagement levels, where the distinct roles of young and old sub-populations diminish or disappear under these challenging conditions. It is worthwhile noting that age-polyethism does not necessarily relate to a higher colony efficiency.

The space of steady-state behaviours can be divided into two main regions:

**Regulation failure:** In this case, all individuals, regardless of age, develop towards full engagement in only one of the tasks, neglecting the other one. The brown region shows this behaviour. It is characterised by very low *b*-values, i.e. extremely scare environments in which foraging is very hard.

**Population/Sub-population branching:** In all other cases, the population branches, i.e. the colony splits into two groups, one fully engaged in foraging (Task 0) with the other entirely engaged in thermoregulation (Task 1). The green/blue region of Fig 5 shows this behaviour for varying levels of *Relative colony efficiency*.

However, depending on the parameters, these subpopulations have different characteristics: The subpopulation dedicated to any one task can either be composed of a mix of both age groups or exclusively/predominantly constituted by a single age group. The latter case captures the emergence of age-polyethism. Cases (c) and (d) in the green region of Fig 5 shows this behaviour. For the parameters investigated, which reflect that foraging (Task 0) is easier for older individuals, young individuals move towards thermoregulation, as expected, while old individuals develop towards foraging.

**Fig 6:**
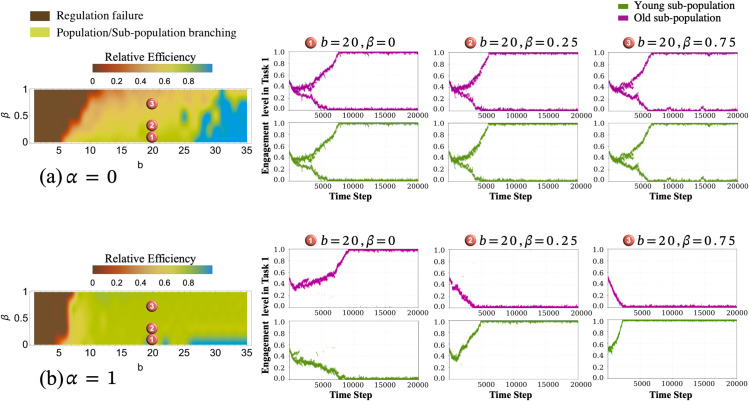
Simulation results of the system for a) α = 0 and b) α = 1 in the branching behavioural regions when varying β and b parameters. **a)** Population branching behaviour: *b* = 20, and β∈{0,0.25,0.75} where both young and old sub-populations divide between the two tasks; **b)** Age specialization behaviour: *b* = 20, and β∈{0,0.25,0.75}.

The above analysis was based on an intermediate imitation/recruitment strength between age groups (α=0.5). Next, we ask how the *SL* model behaves in the limit of α=0andα=1 (Fig 6). We see that if imitation/recruitment between age groups in unrestricted, the population branches into task groups of mixed age (Fig 6a); whereas each task group is exclusively constituted by a single age group if imitation/recruitment between age groups is strongly restricted (Fig 6b).

In each of these settings, *β* determines how the population splits. At β=0, the cost of both tasks are equal for young and old individuals and the relative cost of foraging (Task 0) increases for young individuals with increasing *β*. Thus, for α=1, with increasing *β*, age specialisation occurs in such a form that the group engaging in Task 0 (foraging) predominantly consists of old individuals, whereas young individuals engage preferentially in Task 1.

Beyond showing that age polyethism will not emerge under certain environmental conditions, the simulations also confirm that existing polyethism (as a starting condition) can collapse under these conditions. This is confirmed by the theoretical analysis in Appendix 2 using adaptive dynamics. Small isolated regions of unusually high or low efficiency (visible as ’islands’) in the heatmaps of colony efficiency are likely due to stochastic fluctuations amplified by the nonlinearity of task allocation dynamics. These features do not represent stable behavioural regimes but emerge sporadically under sensitive parameter settings.

Technically, the collapse of the age specialisation with increasing environment pressure (decreasing *b*) as illustrated in Fig 5 can be explained as follows: The game theoretical component of the model captures social learning/recruitment, and the threshold component captures the fact that the colony must satisfy colony requirements. The emergence of age-polytheism is driven by the social learning and manifests in EGT as branching [42,46–48]. The branching, however, is moderated by the stimulus-response mechanism. In parameter regions with very high environmental pressure (low *b*) and thus high task demand for foraging a divided population would be unable to satisfy task demands successfully so that the branching is suppressed. In these regions, the high task demand for foraging forces the younger sub-population to participate in the more costly foraging task in an effort to contribute to satisfying the global colony requirements.

## 4. Discussion

Effective division of labour (DOL) is a crucial aspect of social insect colonies, as it helps to increase their efficiency in the presence of multiple tasks necessary for the survival of the colony. DOL often occurs in the form of age-polyethism, where individuals change their task sets as they age, for example, prominently in honey bees [5]. This often relates to a location preference in the colony which determines the available tasks [49].

Hormonal changes throughout the life of the individual have been identified as an important factor for some forms of age-polyethism with empirical studies establishing that hormonal fluctuations play a role in determining the tasks an individual will specialize in [14,15]. However, it has also been found that the age at which individuals begin to differentiate their tasks is not affected by hormonal changes [16]. This suggests that other factors may also play a role in the division of labour. Changes at the molecular and physiological levels that may alter the individual’s abilities and preferences have also been highlighted as potential determinants of the onset age for task switching [50–52]. Importantly, other factors that can influence the onset of task differentiation include the level of pollen and nectar stores in the colony [53]. If the colony is experiencing a shortage of food, the individuals may switch tasks to focus on foraging and food collection. In other words, dynamically changing task demands can have an impact on age-related task differentiation. Understanding the potential mechanisms for this is crucial for a complete understanding of age-polyethism and, ultimately, the survival and success of social insect colonies. Despite the interest in this topic, only a limited amount of modelling work has been done to identify the underlying causes and general principles involved in age-polyethism [10,13].

One hypothesis that has been widely debated is the idea that social interactions play a crucial role in determining task allocation [54–56]. Accordingly, individuals can adjust their behaviour based on their interactions with others and their environment. For example, individuals may change their behaviour depending on who they interact with, where they interact, and what type of interaction they are involved in [19,57–59]. Generally, individuals can benefit from copying others [60], and in bumblebees and some ant species, individuals have been observed to copy the behaviour of their colony members, which can increase the efficiency of the colony as a whole [25,61–64].

Given the importance of social interactions in shaping the behaviour of individuals, the question suggests itself whether these interactions could be involved in the emergence of age-polyethism. Our analysis shows that age-polyethism can emerge if individuals are more readily entrained by and/or preferential imitate the behaviour of others they are similar to in age. This leaves the question how individuals would make this distinction. Two possibilities immediately come to mind: Firstly, morphological similarities could form the basis of an (indirect) age differentiation. Given that some morphological aspects and specifically cuticular hydrocarbons (CHC) profiles in insects correlate with age [65,66], age polyethism could emerge if individuals are more likely to follow a recruitment signal from individuals similar to themselves. Secondly, it has been known for a long time that younger individuals typically perform in-nest tasks while out-of-nest tasks are preferentially performed by older individuals [54,67]. The differentiation in recruitment probabilities due to more frequent encounters within the same spatial context alone could underpin the above-described dynamics. While this would require an initial spatial separation to have arisen for other reasons, the self-reinforcing dynamics of the recruitment process could amplify even weak and possibly fluctuating initial spatial preference into full and stable age-polyethism.

We note that while a binary age division allows for analytical and computational tractability in our proposed model, real colonies exhibit more nuanced age structures. Such nuanced and continuous age representation could introduce gradual transitions in task allocation and recruitment dynamics, rather than sharp group-based differentiation. However, we expect the core findings of our model to be robust to such generalizations. The mechanism driving age-polyethism in our framework—namely, similarity-modulated social learning combined with environmental task demands—should naturally extend to continuous age spaces. For example, imitation probabilities could smoothly vary with age distance, and specialization could emerge along a spectrum rather than between distinct groups.

Our model assumes that out-of-nest tasks, like foraging, provide increasing marginal benefits and are typically less costly for older individuals, while in-nest tasks, such as brood care or thermoregulation, have diminishing returns and are less age-dependent. We use cost and benefit functions that capture these qualitative differences. The imitation dynamics are modulated by age-similarity and payoff differences, meaning that coordination emerges from decentralized individual-level learning. Importantly, while this minimal model simplifies real-world task structures, it preserves essential features needed to investigate the emergence of age-polyethism. Future extensions could explore more complex task taxonomies or continuous age distributions.

One of the most commonly used approaches in modelling DOL in social insects are response threshold-based models. These models are based on the idea that individuals have a threshold for performing certain tasks, and that as they age, their thresholds for different tasks change, leading to specialization in certain tasks. While these models are useful in explaining some of the empirical patterns of division of labour, they have limitations. Particularly, one main limitation of response threshold-based models is that they cannot explicitly account for the impact of individual interactions. In this paper, we have thus proposed a new integrated model for task selection in social insect colonies that combines response thresholds with an interaction model. This model accounts for both social interactions and environmental factors, providing an alternative explanation of (some aspects) of age-polyethism.

One of the key findings from our model is that social learning can lead to the emergence of age-polyethism. Our model also confirms recent empirical findings [8,68–70], which suggest that age-polyethism does not necessarily lead to optimal colony efficiency, providing a theoretical underpinning for these empirical observations. This is the result of coordination being orchestrated only by independent individual signals. Individuals have to regulate their task engagement based solely on immediately accessible local information that they can directly perceive and on interactions with peers. As game theory teaches us, following only such individual-level feedback does in general not lead to optimality for the collective. Under which specific conditions individual-level feedback leads to collective optimality is indeed a core topic of game-theoretic research [29].

The theory that age polyethism increases colony efficiency in regards to particular work output seem to start with Seeley [67]. However, later experimental work puts the generality of this assumptions in question. Direct experimental work on the efficiency of age polyethism is sparse, likely because this aspect of colony life is difficult to manipulate: while age groups can be culled, tasks cannot be reassigned. The function of age polyethism can thus not be isolated experimentally. What can be studied is how a colony set up for age polyethism changes its function under a changed age structure. In the specific context of honey bees, Rueppell et al. [69] experimentally assembled hives composed of only one age cohort, two distinct age cohorts, or a natural, continuous age distribution measuring worker lifespan and brood production. Colonies with a natural age structure did not consistently produce more brood or have longer-lived workers than the manipulated colonies. None of the treatments reliably maximized longevity and productivity. The authors conclude that the normal age distribution is not a special colony-level adaptation, but rather an emergent outcome of individual life-history trade-offs. Equivalent insights were gained in experiments with *Pheidole dentate* [8] that identified older workers as more efficient in brood care than the younger workers who specialise in this task. Along similar lines, Giehr et al. [70] manipulated age structure in colonies of the ant *Cardiocondyla obscurior* that exhibits age polytheism. The authors formed colonies consisting of only young or only old workers (with corresponding queens). All colonies remained functional and allowing an age structured division did not boost productivity. They conclude that age demography was not required for high colony performance.

More generally, there is mounting empirical evidence that specialisation does not always equate to higher performance (reviewed in [71]). For example, Dornhaus [72] compared individual performance of *Temnothorax albipennis* ant workers between specialists and generalists in various tasks (foraging, nest building, brood transport). They found that specialist workers were not more efficient and that the division of labour did not increase individual work performance. Santoro et al.’s studies revealed that specialisation can even decrease individual performance. Investigating foraging in the social wasp *Vespula vulgaris*, they found that specialised foragers made fewer successful trips per day, spent longer per trip and died younger [68].

However, such studies do not necessarily contradict the assumption that age polyethism increases colony fitness. This is because the above studies assess efficiency in relatively simple, directly measurable factors - as they must. Most importantly, they assess the task performance at the level of individuals. The basis of an extrapolation to colony performance is the assumption that individual task execution benefits are cumulative across the colony. This established approach has also underpinned our theoretical study of colony performance. However, such an extrapolation may not capture other factors that influence the performance at the colony level. Colony fitness may well be a much more complex aggregate of multiple interacting factors. Most importantly, colony fitness may depend not only on individual performance, but also on the resilience of the colony as a whole. This has also been pointed out in a recent review [73]. A more complex concept of fitness may thus well increase even when more direct efficiency measures decrease.

One important and clearly identifiable factor that surely must enter into colony fitness is the survival rate of individuals, and this appears to be strongly impacted by age structures. Taking the survival into account does indeed show a different picture: Perry et al. [10] induced precocious foraging by skewing colony demography and tracked the foragers with RFID tags. Precocious foragers completed far fewer foraging trips and died sooner than normal-age foragers. A demographic model showed that chronic early foraging creates a vicious cycle of loss, leading to rapid colony decline: young bees forage, die, then even younger bees must forage, etc., ultimately breaking down the division of labour. Thus accelerating the age-polyethism schedule destabilized colony performance and induced colony failure. Similar findings come from the comparison of field hives near pesticide-contaminated sites to controls [30] that found that pesticide exposure induced precocious task transitions, disrupting normal age polyethism, and resulted in reduced worker longevity. The importance of mortality rates is also backed up by theoretical models that show that caste polyethism can be more advantageous than age polyethism unless the difference in mortality rates is high in which case the reverse it true [74].

In line with this, theoretical studies in evolutionary biology have likewise found “that division of labour is not merely a group-level adaptation that evolves to maximize group efficiency” [75].

Recent research consistently suggests that colonies may trade short-term task efficiency for stability and resilience. A more complete concept of colony efficiency should therefore account for how age polyethism—and division of labour more broadly—contributes to colony resilience.

Most crucially, our model shows that the division of labour can be reshaped by environmental stressors and that high environmental pressures can lead to a collapse of age-polyethism. This is consistent with the empirical phenomenon reported and analysed in [10], and our model provides a plausible explanation for detailed proximate mechanisms that can lead to this.

Our model does not explicitly account for the increased risks (and thus the higher mortality rate) that precocious foragers can suffer. Extending the model to account for mortality would be an interesting next step. The logical extrapolation of our model is that this would extend the parameter region of inviability as even colonies that may still just be able to satisfy task demand by using younger workers as foragers would become inviable if too many of these are lost.

Further empirical studies to isolate the effects of varying environmental conditions and task difficulties on task preferences are needed to ground our understanding of the role of social learning in task selection. We have begun to explore this in *Iridomyrmex suchieri*. The central assumption of our paper is that individuals are more likely to be entrained by or to copy the behaviour of other individuals that are more similar to. This is empirically testable. It would, for example, be straightforward to extend the social learning experiments described in [76] by controlling and accounting for individual similarity.

Notwithstanding the need for empirical studies, we believe that our theoretical approach provides plausible evidence that the role of social learning in the emergence of age-polyethism deserves further study and that a game-theoretic approach has the potential to elucidate this complex and dynamic phenomenon.

## Supporting information

Appendix 1 Simulation parameters, benefit and cost functions. (PDF)

Appendix 2 Evolution of population traits. (PDF)
