# Supplementary material for: Age polyethism can emerge from social learning: A game-theoretic investigation
Source: PLoS Comput Biol. 2025 Aug 25;21(8):e1013415. doi: 10.1371/journal.pcbi.1013415 (PMC12396761; doi:10.1371/journal.pcbi.1013415)
Supplement: Appendix 1 — (PDF) [file pcbi.1013415.s001.pdf]

# Supplementary Information

# Age Polyethism Can Emerge from Social Learning: A Game-Theoretic Investigation

Moein Khajehnejad[1,2,*], Julian García[1], Bernd Meyer[1]

[1]Department of Data Science and Artificial Intelligence, Faculty of Information Technology, Monash University, Clayton, Victoria, Australia
[2]Turner Institute for Brain and Mental Health, Monash University, Clayton, Victoria, Australia
[*] Corresponding author: moein.khajehnejad@monash.edu

## Appendix 1: Simulation Parameters, Benefit and Cost Functions

S1 Table summarizes the selected parameter values for the simullation results reported in main text.

S1 Table: Parameters of the simulations

| Notation | Definition | Values |
|---|---|---|
| $b$ | Quadratic coefficient of benefit function in thermoregulation task | (1,40) |
| $\beta$ | The cost difference of engaging in Task 0 for different age groups | (0,1) |
| $r$ | The cost ratio of Tasks 1 and 0 | 0.5 |
| $N$ | Total population size | 500 |
| $n$ | Group size that individual interactions are restricted to | 10 |
| $\mu$ | Probability with which an individual explores a new response trait (mutation rate) | 0.1 |
| $\sigma$ | Standard deviation of the Gaussian distribution around trait value | 0.005 |
| $\alpha$ | Strength of restriction in recruitment from other age groups during imitation | (0,1) |
| $\omega$ | Tuning parameter to keep the effect of age similarity index from being strongly damped | 10 |
| $\eta$ | Learning rate in the Individual Gradient-based paradigm | 0.005 |
| $\lambda_1$ | Bounding parameter to keep $S_0$ in unit interval | $0.5/B_{0_{min}}$ |
| $\lambda_2$ | Saturating parameter to prevent $S_0$ from growing to infinity | 0.8 |
| $\phi_1$ | Bounding parameter to keep $S_1$ in unit interval | 1 |
| $\phi_2$ | Saturating parameter to prevent $S_1$ from growing to infinity | 0.8 |
| $\gamma$ | Growing slope of action probability | 10 |

At any time step, the state of the colony with size $N$ can be determined by a 2-dimensional vector $(x_j, a_j)_{j=1,\cdots,N}$, where $x_j$ shows the engagement level to thermoregulation task (i.e. $1 - x_j$ represents the engagement in the foraging task) and $a_j$ is the age group of the individual $j$. $a_j = 0$ indicates the young fraction of the population where $a_j = 1$ stands for the old sub-population. Here, the interactions among individuals are limited to groups of size $n$, where we have $K = \frac{N}{n}$ groups or games in the colony. Thereby, in every game, each individual's payoff depends not only on their own trait values but also on the collective behaviour of other players in that game. Particularly, game $G_k$, $k \in \{1, \cdots, K\}$ is given by $(\mathcal{X}_k, \mathcal{A}_k)$; where $\mathcal{X}_k = \{x_i\}_{i \in G_k}$ and $\mathcal{A}_k = \{a_i\}_{i \in G_k}$ are trait vectors and age group vectors respectively.

The payoff for individual $j$ in game $G_k$, is:

$$\Pi_{j,G_k} = B(\mathcal{X}_k) - C(x_j, a_j), \tag{11}$$

where $B(\mathcal{X}_k)$ is the total benefit in game $G_k$ which is shared evenly among $n$ individuals and $C(x_j, a_j)$ reflects the individual costs for the worker $j$. The simultaneous modulation of the colony's temperature and food resources is required for the colony to survive and thrive. Hence, we represent the total benefit function as multiplicative between the two task benefits. Costs are not shared among the players in the game. We define the total benefit inside each game and the total costs for each individual as:

$$B(\mathcal{X}_k) = \frac{1}{n}\, B_T(\sum_{i \in G_k} x_i) \cdot B_F(\sum_{i \in G_k} x_i), \tag{12}$$

$$C(x_j, a_j) = C_0(x_j, a_j) + C_1(x_j). \tag{13}$$

Now, let us represent the benefit and cost functions associated with both tasks present in the environment with $B_0(\cdot), B_1(\cdot)$ and $C_0(\cdot), C_1(\cdot)$. For each game $G_k$ we have:

$$B_0(\sum_{i \in G_k} x_i) = b \cdot \big( \sum_{i \in G_k} (1 - x_i) \big), \tag{14}$$

$$C_0(x_j, a_j) = \big( - (1 - x_j)^2 + 2\,(1 - x_j) \big) \cdot exp(\beta\,(1 - a_j)), \tag{15}$$

$$B_1(\sum_{i \in G_k} x_i) = -\frac{4}{n^2}\,\big( \sum_{i \in G_k} x_i \big)^2 + \frac{4}{n}\,\big( \sum_{i \in G_k} x_i \big), \tag{16}$$

$$C_1(x_j) = r \cdot x_j, \tag{17}$$

where the cost of the foraging task $C_0(x_j, a_j)$ is age-dependent, meaning that individuals from different age groups will experience different cost of different magnitudes when engaging equally with a task. In this context, the foraging task tends to be more costly for the young population at equal levels of engagement due to the higher risks of out-nest tasks such as foraging for inexperienced young workers. The specific shapes of $B_i$ and $C_i$ are chosen according to earlier literature [77] to represent the combination of a maximising task, such as foraging, with a homeostatic task, such as thermoregulation.
