# Supplementary material for: Age polyethism can emerge from social learning: A game-theoretic investigation
Source: PLoS Comput Biol. 2025 Aug 25;21(8):e1013415. doi: 10.1371/journal.pcbi.1013415 (PMC12396761; doi:10.1371/journal.pcbi.1013415)
Supplement: Appendix 2 — (PDF) [file pcbi.1013415.s002.pdf]

Appendix 2: Evolution of Population Traits

Single population without age difference:

In the first case all individuals can imitate one another. This means every individual (probabilistically) imitates the player with the highest payoff value. To understand how the strategy distribution of individuals changes via imitating the successful ones followed by small exploring (mutations) in the strategy space, we exploit adaptive dynamics [42, 43] as a mathematical framework using an infinite population to give a deterministic approximation of the stochastic dynamics of an EGT model.
We start by defining the invasion fitness function for a potential mutant x' which is nearby the resident population with trait x. $\mathcal{F}_x(x')$ represents this fitness value. Here, without loss of generality we assume the population only consists of *old* individuals.

$$\mathcal{F}_x(x') = \Pi\big(x'; (n-1)x\big) - \Pi\big(x; (n-1)x\big), \tag{18}$$

where $\Pi(x'; (n-1)x)$ is the payoff function for an individual with trait value x' in a game with $n-1$ other players having trait values summing up to $(n-1)x$. We now have:

$$\begin{aligned}\Pi\big(x'; (n-1)x\big) &= \frac{1}{n}B_0\big(x' + (n-1)x\big) \cdot B_1\big(x' + (n-1)x\big) - \big(C_0(x', 1) + C_1(x')\big)\\ &= \frac{1}{n}\big(- \frac{4}{n^2}(x' + (n-1)x)^2 + \frac{4}{n}(x' + (n-1)x)\big) \cdot b(n - x' - (n-1)x) - \big(rx' - (1-x')^2 + 2(1-x')\big).\end{aligned}$$

Thus, the selection gradient for the old population is defined as:

$$\begin{aligned}\mathcal{D}(x) &= \frac{\partial \mathcal{F}_x(x')}{\partial x'}\Big|_{x'=x}\\ &= \frac{1}{n}B_0'\big(nx\big) \cdot B_1\big(nx\big) + \frac{1}{n}B_0\big(nx\big) \cdot B_1'\big(nx\big) - C_0'(x, 1) - C_1'(x).\end{aligned}$$

Then, the solution of the following gives us the singular strategies (x^*):

$$\mathcal{D}(x^*) = \frac{1}{n}B_0'\big(nx^*\big) \cdot B_1\big(nx^*\big) + \frac{1}{n}B_0\big(nx^*\big) \cdot B_1'\big(nx^*\big) - C_0'(x^*, 1) - C_1'(x^*) = 0. \tag{19}$$

If $x^* \in [0, 1]$ exists, this singular strategy is guaranteed to be convergent stable and satisfies the necessary conditions for both Evolutionary Branching and Evolutionary Stable Strategies if the below condition holds:

$$\frac{d\mathcal{D}(x)}{dx}\Big|_{x=x^*} = B_0''\big(nx^*\big) \cdot B_1\big(nx^*\big) + B_0\big(nx^*\big) \cdot B_1''\big(nx^*\big) + 2B_0'\big(nx^*\big) \cdot B_1'\big(nx^*\big) - C_0''(x^*, 1) - C_1''(x^*) < 0. \tag{20}$$

Moreover, in a population that is attracted to the convergent stable strategy, the curvature of invasion fitness at this point determines the subsequent evolutionary fate. Indeed, x^* is an evolutionary stable strategy (ESS) and the population cannot be invaded by any nearby rare mutants, if $\mathcal{F}_x(x')$ takes its maximum value at x^*. However, if it takes the minimum value at x^*, the population can be invaded by proximate mutants on either side of the singular point. Hence, x^* is the Evolutionary Branching Strategy. In summary, the population becomes generalized, if:

$$\frac{\partial^2 \mathcal{F}_{x^*}(x')}{\partial x'^2}\Big|_{x'=x^*} = \frac{1}{n}B_0''\big(nx^*\big) \cdot B_1\big(nx^*\big) + \frac{1}{n}B_0\big(nx^*\big) \cdot B_1''\big(nx^*\big) + \frac{2}{n}B_0'\big(nx^*\big) \cdot B_1'\big(nx^*\big) - C_0''(x^*, 1) - C_1''(x^*) < 0, \tag{21}$$

and it branches if:

$$\frac{\partial^2 \mathcal{F}_{x^*}(x')}{\partial x'^2}\Big|_{x'=x^*} = \frac{1}{n}B_0''\big(nx^*\big) \cdot B_1\big(nx^*\big) + \frac{1}{n}B_0\big(nx^*\big) \cdot B_1''\big(nx^*\big) + \frac{2}{n}B_0'\big(nx^*\big) \cdot B_1'\big(nx^*\big) - C_0''(x^*, 1) - C_1''(x^*) > 0. \tag{22}$$

Strong separation of age groups for imitation ($\alpha = 1$):

In the second scenario, age groups are highly restricted to imitate same-age individuals, i.e. $\alpha = 1$. Individuals select successful players from their own age group to imitate. The invasion fitness of an old (young) mutant with trait value x'

(or y') in the resident population where old individuals have trait x and young ones have trait y is:

$$\mathcal{F}^{old}_{x,y}(x') = \sum_{i=0}^{n-1} \binom{n-1}{i} \rho^i (1-\rho)^{n-1-i} \Big(\Pi^O\big(x'; (i)x + (n-1-i)y\big) \Big),$$

$$\mathcal{F}^{young}_{x,y}(y') = \sum_{i=0}^{n-1} \binom{n-1}{i} \rho^i (1-\rho)^{n-1-i} \Big(\Pi^Y\big(y'; (i)x + (n-1-i)y\big) \Big),$$

where ρ represents the fraction of old individuals in the population.
The payoff for an individual with trait x' from the old/young age group (Π^O/Π^Y) is then defined as:

$$\Pi^O\big(x'; (i)x + (n-1-i)y\big) = \frac{1}{n} B_0\big(x' + (i)x + (n-1-i)y\big) \cdot B_1\big(x' + (i)x + (n-1-i)y\big) - \big(C_0(x', 1) + C_1(x')\big),$$
$$= \tfrac{1}{n}\big(-\tfrac{4}{n^2}(x' + i\,x + (n-1-i)y)^2 + \tfrac{4}{n}(x' + i\,x + (n-1-i)y)\big) \cdot b(n - x' - i\,x - (n-1-i)y) - \big(rx' - (1-x')^2 + 2(1-x')\big).$$

$$\Pi^Y\big(y'; (i)x + (n-1-i)y\big) = \frac{1}{n} B_0\big(y' + (i)x + (n-1-i)y\big) \cdot B_1\big(y' + (i)x + (n-1-i)y\big) - \big(C_0(y', 0) + C_1(y')\big)$$
$$= \tfrac{1}{n}\big(-\tfrac{4}{n^2}(y' + i\,x + (n-1-i)y)^2 + \tfrac{4}{n}(y' + i\,x + (n-1-i)y)\big) \cdot b(n - y' - i\,x - (n-1-i)y) - \big(ry' + (-(1-y')^2 + 2(1-y'))e^{\beta}\big)$$

Next, we have the selection gradient for old and young populations as follows:

$$\mathcal{D}^{old}(x,y) = \frac{\partial \mathcal{F}^{old}_{x,y}(x')}{\partial x'}\Big|_{x'=x} =$$
$$\tfrac{1}{n}B_0'\big((i+1)x + (n-1-i)y\big) \cdot B_1\big((i+1)x + (n-1-i)y\big) + \tfrac{1}{n}B_0\big((i+1)x + (n-1-i)y\big) \cdot B_1'\big((i+1)x + (n-1-i)y\big) - \big(C_0'(x,1) + C_1'(x)\big),$$

$$\mathcal{D}^{young}(x,y) = \frac{\partial \mathcal{F}^{young}_{x,y}(y')}{\partial y'}\Big|_{y'=x} =$$
$$\tfrac{1}{n}B_0'\big(ix + (n-i)y\big) \cdot B_1\big(ix + (n-i)y\big) + \tfrac{1}{n}B_0\big(ix + (n-i)y\big) \cdot B_1'\big(ix + (n-i)y\big) - \big(C_0'(y,0) + C_1'(y)\big).$$

Then, the solution of the following gives us the singular coalition point (x^*, y^*):

$$\mathcal{D}^{old}(x^*, y^*) = \mathcal{D}^{young}(x^*, y^*) = 0. \tag{23}$$

Assuming the singular coalition point ($x^*, y^* \in [0,1]$) exists, it is evolutionarily stable (meaning no new mutants can invade the population) if, and only if, x^* and y^* are ESS:

$$\frac{\partial^2 \mathcal{F}^{old}_{x,y}(x')}{\partial x'^2}\Big|_{\substack{x'=x^*\\ y=y^*}} < 0, \quad \frac{\partial^2 \mathcal{F}^{young}_{x,y}(y')}{\partial y'^2}\Big|_{\substack{x=x^*\\ y'=y^*}} < 0.$$

Moreover, *invadability* by rare proximate mutants near a singular coalition point can happen, if and only if:

$$\frac{\partial^2 \mathcal{F}^{old}_{x,y}(x')}{\partial x^2}\Big|_{\substack{x'=x^*\\ y=y^*}} < -\frac{\partial^2 \mathcal{F}^{old}_{x,y}(x')}{\partial x'^2}\Big|_{\substack{x'=x^*\\ y=y^*}}$$
$$,$$
$$\frac{\partial^2 \mathcal{F}^{young}_{x,y}(y')}{\partial y^2}\Big|_{\substack{x=x^*\\ y'=y^*}} < -\frac{\partial^2 \mathcal{F}^{young}_{x,y}(y')}{\partial y'^2}\Big|_{\substack{x=x^*\\ y'=y^*}}.$$

Whether a singular coalition (x^*, y^*) is convergent stable or not can be determined by the linear stability analysis around (x^*, y^*). To do so, the attractivity of the singular coalition is specified by the sign of eigenvalues in the Jacobian matrix ($\mathbf{J}$) of the selection gradients:

$$\mathbf{J} = \begin{pmatrix} \frac{\partial \mathcal{D}^{old}(x,y)}{\partial x}\big|_{\substack{x=x^*\\ y=y^*}} & \frac{\partial \mathcal{D}^{old}(x,y)}{\partial y}\big|_{\substack{x=x^*\\ y=y^*}} \\ \frac{\partial D^{young}(x,y)}{\partial x}\big|_{\substack{x=x^*\\ y=y^*}} & \frac{\partial D^{young}(x,y)}{\partial y}\big|_{\substack{x=x^*\\ y=y^*}} \end{pmatrix}.$$

Assuming λ_1 and λ_2 are the eigenvalues of matrix $\mathbf{J}$, the singular coalition (x^*, y^*) is an attractor of the evolutionary dynamics (convergent stable) if both λ_1 and λ_2 have negative real parts ($\Re\{\lambda_1\}, \Re\{\lambda_2\} < 0$) and repelling (unstable) if at least one eigenvalue has positive real part ($\Re\{\lambda_1\} \cdot \Re\{\lambda_2\} > 0$).

Invadability cannot happen if the strategy is simultaneously evolutionary stable. A singular coalition point that is convergent stable but for which at least one of x^* or y^* does not meet the ESS stability criteria while satisfying nearby invadibility, will lead to further branching of the strategies.

Analysis of dimorphic population evolution past the branching point for $0 \leq \alpha \leq 1$:

Let us consider having a dimorphic population with trait values x and y where ρ is the fraction of the young population and $(1-\rho)$ represents the old population fraction. Now, ρ_x^Y and ρ_x^O show the fraction of young and old populations with trait value x respectively. The average payoffs for each sub-population of young or old (with different trait values, x or y) are as follows:

$$\Pi_x^Y = \sum_{i=0}^{n-1} \binom{n-1}{i} \rho^i (1-\rho)^{n-1-i} \Big[\sum_{j=0}^{i} \sum_{k=0}^{n-1-i} \binom{i}{j} \binom{n-1-i}{k} \cdot (\rho_x^Y)^j \cdot (1-\rho_x^Y)^{i-j} \cdot (\rho_x^O)^k \cdot (1-\rho_x^O)^{n-1-i-k} \cdot \Pi^Y\big(x; (k+j)x + (n-1-k-j)y\big) \Big],$$
$$\Pi_y^Y = \sum_{i=0}^{n-1} \binom{n-1}{i} \rho^i (1-\rho)^{n-1-i} \Big[\sum_{j=0}^{i} \sum_{k=0}^{n-1-i} \binom{i}{j} \binom{n-1-i}{k} \cdot (\rho_x^Y)^j \cdot (1-\rho_x^Y)^{i-j} \cdot (\rho_x^O)^k \cdot (1-\rho_x^O)^{n-1-i-k} \cdot \Pi^Y\big(y; (k+j)x + (n-1-k-j)y\big) \Big],$$
$$\Pi_x^O = \sum_{i=0}^{n-1} \binom{n-1}{i} \rho^i (1-\rho)^{n-1-i} \Big[\sum_{j=0}^{i} \sum_{k=0}^{n-1-i} \binom{i}{j} \binom{n-1-i}{k} \cdot (\rho_x^Y)^j \cdot (1-\rho_x^Y)^{i-j} \cdot (\rho_x^O)^k \cdot (1-\rho_x^O)^{n-1-i-k} \cdot \Pi^O\big(x; (k+j)x + (n-1-k-j)y\big) \Big],$$
$$\Pi_y^O = \sum_{i=0}^{n-1} \binom{n-1}{i} \rho^i (1-\rho)^{n-1-i} \Big[\sum_{j=0}^{i} \sum_{k=0}^{n-1-i} \binom{i}{j} \binom{n-1-i}{k} \cdot (\rho_x^Y)^j \cdot (1-\rho_x^Y)^{i-j} \cdot (\rho_x^O)^k \cdot (1-\rho_x^O)^{n-1-i-k} \cdot \Pi^O\big(y; (k+j)x + (n-1-k-j)y\big) \Big].$$

Now using replicator dynamics, we obtain the dynamics of ρ_x^Y and ρ_x^O according to α.

$$\frac{d\rho_x^Y}{dt} = \rho_x^Y (1-\rho_x^Y) \cdot \Big[\frac{\rho_x^Y \cdot \Pi_x^Y + (1-\alpha) \cdot \rho_x^O \cdot \Pi_x^O}{\rho_x^Y + (1-\alpha) \cdot \rho_x^O} - \frac{\rho_y^Y \cdot \Pi_y^Y + (1-\alpha) \cdot \rho_y^O \cdot \Pi_y^O}{\rho_y^Y + (1-\alpha) \cdot \rho_y^O} \Big],$$
$$\frac{d\rho_x^O}{dt} = \rho_x^O (1-\rho_x^O) \cdot \Big[\frac{\rho_x^O \cdot \Pi_x^O + (1-\alpha) \cdot \rho_x^Y \cdot \Pi_x^Y}{\rho_x^O + (1-\alpha) \cdot \rho_x^Y} - \frac{\rho_y^O \cdot \Pi_y^O + (1-\alpha) \cdot \rho_y^Y \cdot \Pi_y^Y}{\rho_y^O + (1-\alpha) \cdot \rho_y^Y} \Big].$$

The system's steady state is then determined as the joint solution of $\frac{d\rho_x^Y}{dt} = 0$ and $\frac{d\rho_x^O}{dt} = 0$.

This completes the analytical confirmation of the simulation results given in Section 3.1.
